# Menopause following cancer treatment-induced menopause: unique considerations and current recommendations

**DOI:** 10.1530/RAF-26-0082

**Published:** 2026-10-01

**Authors:** Gabriella Yongue, Zachary Nash, Kristyn Manley, Melanie C Davies, Shibani Nicum

**Affiliations:** ^1^ University College London Cancer Institute, London, United Kingdom; ^2^ University College London Hospital, London, United Kingdom; ^3^ EGA Institute for Women’s Health, London, United Kingdom; ^4^ University Hospitals Bristol NHS Foundation Trust, Bristol, United Kingdom

**Keywords:** premature ovarian insufficiency, iatrogenic menopause, cancer treatment, cancer survivorship, holistic management

## Abstract

**Abstract:**

Menopause following cancer treatment is an increasingly recognised component of survivorship yet remains under-addressed in clinical care. The aim of this review was to synthesise current evidence on menopause induced by cancer treatment, by bringing together current evidence on the underlying mechanisms, clinical manifestations, and management in the context of survivorship care. Evidence was evaluated from contemporary clinical studies, guideline recommendations, and emerging evidence across oncology and menopause research. Cancer treatment-induced menopause commonly results from surgical removal of the ovaries, follicular depletion from chemotherapy, and radiation-induced ovarian damage, and in some cases, it is chemically induced by gonadotrophin-releasing hormone agonists. The resulting symptom burden is often substantial, including vasomotor symptoms, mood disturbance, genitourinary syndrome, sexual dysfunction, fatigue, and cognitive difficulties that impair quality of life. In addition, premature or early menopause is associated with increased long-term health risks, including reduced bone density, cardiovascular disease, and adverse psychosocial outcomes, such as infertility and altered self-identity. Menopause symptoms can also overlap with treatment side effects, and management is often complex, requiring an individualised, multidisciplinary approach. Whilst hormone replacement therapy is effective, its use is limited by concerns regarding cancer recurrence in hormone-sensitive disease. As a result, non-hormonal and supportive strategies play a pivotal role in symptom management. Despite increasing recognition of the impact of menopause, there are few effective licensed therapies and access to specialist support and counselling is limited. Integrated menopause care that incorporates menopause management into oncology pathways offers a pragmatic approach, providing holistic, patient-centred strategies that balance long-term health risks with quality-of-life priorities.

**Lay summary:**

Cancer treatment, including surgery, chemotherapy, radiotherapy, or long-term therapies, can cause menopause, which is often sudden, resulting in severe symptoms that may overlap with treatment-related side effects. Menopause can therefore be complex to manage, and there may also be added concerns around promoting cancer recurrence. Menopause symptoms can include hot flushes, poor sleep, mood disturbance, vaginal dryness, reduced sexual well-being, fatigue, and problems with memory – these can all impact daily life. There may also be longer-term health impacts, including bone loss and cardiovascular disease. This review brings together current evidence to discuss why treatment-related menopause occurs and how it can be best managed. It highlights that, despite growing awareness, many cancer survivors do not receive adequate information, support, or treatment for these symptoms. As cancer survival improves, menopause care must become a central part of improved survivorship. Personalised approaches to menopause management have the potential to improve long-term health, well-being, and quality of life after cancer.

## Introduction

Menopause marks the end of ovarian endocrine function and is associated with physiological and psychosocial changes. Menopause resulting from cancer treatment, surgery and systemic therapies, is often abrupt in onset and associated with increased severity of symptoms, with increased long-term health impacts in those experiencing an early menopause. Menopause symptoms can also overlap with ongoing cancer treatment side effects, introducing additional management complexity, particularly around the use of menopause hormone replacement therapy, which may impact cancer recurrence risk. This review synthesises current evidence in this area, examines contemporary management strategies, and highlights areas of controversy and ongoing need. By consolidating current perspectives and recommendations, we aim to support clinicians in providing individualised, evidence-based menopause care following cancer treatment.

## Pathophysiology

Cancer treatment-induced menopause is associated with the loss of ovarian function before the expected age of natural menopause. It is classified as premature ovarian insufficiency (POI) if occurring before age 40 and as early menopause (EM) between ages 40 and 44. Globally, the estimated prevalence of POI and EM is 3.7 and 12.2%, respectively (Golezar *et al.* 2019). Although ovarian function may recover in some cases, many experience abrupt and complete ovarian failure leading to more severe symptoms (Marino *et al.* 2014).

Natural menopause usually results in a more gradual endocrine decline, with the postmenopausal ovary continuing to produce androgens, particularly testosterone, that contribute to a small amount of peripheral oestrogen synthesis. Conversely, surgical menopause results in more acute and marked reductions in circulating oestradiol, testosterone, and dehydroepiandrosterone (DHEA) (Wu *et al.* 2025).

Chemotherapy-induced ovarian insufficiency results from damage to the primordial follicle pool, although partial preservation of primordial follicles may allow recovery, particularly in younger women (Meirow *et al.* 2010). The most gonadotoxic drugs are alkylating agents, particularly cyclophosphamide, causing damage to oocytes and granulosa cells in a dose-dependent fashion (Wallace *et al.* 2005). Other drugs, such as platinum agents and doxorubicin, pose a moderate risk of gonadal dysfunction.

Radiotherapy causes ovarian injury through dose-dependent destruction of follicles and vascular compromise. Pelvic irradiation directly exposes the ovaries to ionising radiation and causes menopause within the first six months (Viswanathan *et al.* 2014). Total body irradiation results in ovarian failure in 90% of patients during long-term follow-up (Viswanathan *et al.* 2014).

Endocrine therapies further contribute to hypoestrogenism. Gonadotropin-releasing hormone agonists (GnRHas) induce a reversible hypogonadal state by pituitary desensitisation and suppression of gonadotrophin secretion. This suppresses ovarian steroidogenesis, with full effect achieved within 7–14 days (Conn & Crowley Jr 1991). Aromatase inhibitors suppress oestradiol (E2) and oestrone (E1) production by inhibiting the peripheral conversion of androgens. Letrozole, the most potent agent in this class, reduces circulating oestradiol from a typical postmenopausal range of 10–60 pmol/L to below 1–3 pmol/L (Faltinová *et al.* 2023). They are widely used as adjuvant therapy in oestrogen receptor-positive breast cancer and in selected gynaecological malignancies, including low-grade serous ovarian carcinoma. This profound suppression of oestrogen exacerbates menopausal symptoms and can significantly impact quality of life (Faltinová *et al.* 2023). Tamoxifen is a selective oestrogen receptor modulator with anti-oestrogenic effects on the hypothalamic–pituitary axis. It can cause amenorrhoea and menopause symptoms but without permanently affecting ovarian function. A cohort study demonstrated that, in the absence of chemotherapy, tamoxifen use is not associated with earlier onset of menopause (Chien *et al.* 2015). However, assessment of menopausal status can be challenging, as tamoxifen alters follicle-stimulating hormone (FSH) levels, and therefore, serial hormone measurements may be required (Gradishar *et al.* 2026). Additionally, tamoxifen increases the risk of endometrial hyperplasia and adenocarcinoma, stroke, pulmonary embolism, and embryo-fetal toxicity (National Library of Medicine (NLM), 2021).

The increasing use of cyclin-dependent kinase 4 and 6 (CDK4/6) inhibitors in the management of hormone receptor-positive breast cancer has raised questions regarding their potential impact on ovarian function (Scavone *et al.* 2023). Preclinical data suggest a biological basis for ovarian toxicity, as the cyclin D–CDK4/6 complex plays a key role in granulosa cell proliferation, primordial follicle activation, and oocyte meiosis. However, clinical data remain insufficient and are confounded by the routine co-administration of ovarian suppression in premenopausal women.

## Clinical manifestations

In addition to menopausal symptoms, patients also experience cancer-related symptoms, treatment toxicities, and psychosocial stressors. These factors often intertwine with each other and can exacerbate menopausal symptom severity and negatively impact quality of life. Table 1 summarises the prevalence, severity, and clinical considerations of symptoms, highlighting the unique challenges faced by women experiencing cancer treatment-induced menopause. These symptoms rarely occur in isolation; cancer survivors frequently experience a convergence of symptoms that are driven by both menopausal hormonal changes and cancer treatment-related toxicities. Additionally, fatigue can act as a central amplifier of symptom burden, increasing the severity of hot flashes, mood disturbance, sexual dysfunction, and weight gain (Glaus *et al.* 2006). Understanding how these symptoms occur together and interact is important for individualised menopause management.

**Table 1:** Clinical manifestations of cancer treatment-induced menopause: mechanisms and contributing factors. Summary of key symptom domains, showing the relative contributions of early menopause-related hormonal changes and cancer- or treatment-specific factors.

Symptom domain	Early menopause-related mechanisms	Cancer/treatment-related contributors	Key clinical insights
Vasomotor symptoms	Following risk-reducing oophorectomy, 82% experience symptoms at 12 months without HRT (Hickey *et al.* 2021)	Higher prevalence (79 vs 61%), severity (OR: 2.18), and frequency (OR: 3.61) in cancer survivors (Marino *et al.* 2014)	More severe and frequent; often the most bothersome initial symptom. Possible contraindications to HRT
Genitourinary syndrome and sexual dysfunction	Sexual dysfunction due to surgical menopause can persist despite systemic HRT (Hickey *et al.* 2021)	Surgery, pelvic radiotherapy (vaginal fibrosis), AI, graft-versus-host disease	Under-recognised and undertreated. Vulval dermatoses also more common postmenopause
Psychosocial impact	Hormonal changes affecting libido and body perception	Body image changes, pain, fatigue, psychological distress	Multifactorial; requires biopsychosocial and survivorship-focused care
Mental health and neurocognitive	Menopause <40 y doubles risk of depression (Georgakis *et al.* 2016). Early surgical menopause associated with increased dementia risk (HR: 1.89) (Rocca *et al.* 2007)	Chemotherapy-related cognitive impairment (up to 75%; persistent in ∼33%) (Rovito *et al.* 2025); psychological stress	Overlapping drivers; screen early and manage with supportive, behavioural, and pharmacological strategies
Bone health	Osteoporosis (8–14%) and osteopenia (23–57%) following surgical menopause, premature menopause, or early menopause (Gaba & Manchanda 2020)	Treatment-related bone loss; metastatic disease; AI.	High fracture risk; requires proactive screening (DEXA), prevention, and consideration of bone-protective therapies
Musculoskeletal symptoms	Increased sarcopenia (OR: 2.05) and musculoskeletal discomfort (OR: 2.26) with premature menopause (Vallejo *et al.* 2024)	Taxanes, platinum agents, PARPis, AI (treatment discontinuation up to 30%) (Nardin *et al.* 2025), anti-angiogenics. Higher risk of vitamin D deficiency (Zębalski *et al.* 2025)	Common cause of reduced quality of life and treatment non-adherence; requires symptom-directed management
Cardiometabolic health	POI increases cardiovascular risk (HR: 1.55) (Zhu *et al.* 2020)	Increased cardiovascular (HR: 1.37) and stroke risk (HR: 1.22) (Florido *et al.* 2022); cardiotoxic therapies and AI use	Additive risk; requires early cardiovascular risk assessment with long-term management and follow-up

DEXA, dual-energy X-ray absorptiometry; HR, hazard ratio; HRT, hormone replacement therapy; OR, odds ratio; PARPis, poly (ADP-ribose) polymerase inhibitors; AIs, aromatase inhibitors; POI, premature ovarian insufficiency.

### Menopause spectrum and impact of age at menopause on long-term health risks

Aside from symptom burden, a major concern is accelerated biological ageing and the associated long-term morbidity. Increasing evidence suggests that early loss of ovarian function contributes to biological age acceleration due to epigenetic changes, telomere shortening, and markers of cellular senescence (Zhou *et al.* 2025). In parallel, cancer survivors demonstrate treatment-related biological ageing, with similar alterations linked to increased cardiovascular and metabolic risk (Mandelblatt *et al.* 2021). Therefore, cancer treatment-induced menopause may be conceptualised as a ‘double-hit’ model. Both disruption of the endocrine system and cancer therapies increase biological ageing and long-term morbidity in this population.

A graded relationship is also observed for all-cause mortality across the menopause spectrum. A meta-analysis of seven studies showed that POI is associated with a 40% increased risk of all-cause mortality (RR: 1.40; 95 CI: 1.10–1.77), whilst EM confers a more modest risk (RR: 1.09; 95% CI: 1.00–1.18) (Tao *et al.* 2016). Similarly, there is a 55% higher cardiovascular risk in women with POI, compared with a 30% in those aged 40–44 years relative to the average age of menopause. This gradient is observed in a number of cardiovascular outcomes and persists even after adjustment for other risk factors (Zhu *et al.* 2019). These findings depict a dose–response relationship such that earlier loss of ovarian function is associated with progressively greater adverse health outcomes.

Bone health is also influenced by age at menopause, with earlier ovarian insufficiency associated with a greater risk of low bone density. Data from the Women’s Health Initiative Observational Study (*n* = 21,711) reported that menopause before 40 years was associated with a significantly higher fracture risk and lower bone mineral density (BMD) compared with menopause at age 50 years or older (HR: 1.21; 95% CI: 1.02–1.44) (Sullivan *et al.* 2015). Interventional data demonstrate that this risk is reversible, with hormone replacement therapy (HRT) restoring BMD in women with POI to levels comparable with age-matched controls over 12 months (Popat *et al.* 2014). In the context of cancer treatment-induced menopause, BMD loss is further increased. Population-based data indicate that there is a 10–30% excess fracture risk among cancer survivors compared with the general population (Ye *et al.* 2024).

Given this cumulative risk, early prevention strategies are essential. International guidelines recommend baseline BMD assessment at the time of POI diagnosis (Panay *et al.* 2024). If normal and appropriate hormone replacement therapy (HRT) is initiated, a repeat evaluation within 5 years is not required. However, if osteopenia or osteoporosis is reported, then repeat assessment is advised. Routine advice should include adequate dietary calcium, vitamin D supplementation, and weight-bearing exercise. Where HRT is not recommended, non-hormonal alternatives, such as bisphosphonates, provide comparable benefits in preserving BMD but are associated with different side effect profiles. Zoledronic acid not only improves BMD but also reduces the risk of breast cancer recurrence in postmenopausal women (Early Breast Cancer Trialist’s Collaborative Group 2015). Additionally, cardiovascular risk should be reviewed at diagnosis and monitored yearly, with optimisation of modifiable risk factors including smoking cessation, weight management, physical activity, blood pressure, and metabolic health (lipids and insulin resistance).

Early initiation of HRT in POI, if not contraindicated, can mitigate cardiovascular and bone risk and is advised by international guidelines (Panay *et al.* 2024, National Institute for Health & Care Excellence 2025), although long-term data are limited. Beyond the average age of menopause, decisions regarding continuation of HRT should be individualised, using the lowest effective dose with regular review.

### Fertility, identity, and psychosexual impact of premature ovarian insufficiency

Infertility following a diagnosis of POI is one of the most distressing outcomes for women, particularly those who have not completed their family. Furthermore, the loss of reproductive choice along with the disruption of anticipated life trajectories contributes to the psychological burden. In a cross-sectional survey of 136 women with POI, including women with menopause as a result of cancer treatment, fertility was the most frequently reported specific concern (75%) (Singer *et al.* 2011).

The impact of cancer treatment-induced menopause also extends to broader aspects of identity and self-perception. In the same cohort, 78% of women reported that POI negatively affected their self-image and confidence. Menopause occurring earlier than expected may create a perceived mismatch between chronological age and bodily ageing, contributing to feelings of social isolation from peers who have not yet experienced menopause (Parton *et al.* 2017). Qualitative studies suggest that reproductive capacity remains closely linked to perceptions of femininity, thus contributing to the psychological impact of infertility and menopause (Parton *et al.* 2017).

Sexual dysfunction is also highly prevalent and multifactorial. It can include the loss of libido, vaginal dryness, dyspareunia, and diminished sexual confidence. These symptoms can negatively affect intimate relationships and are often compounded by fatigue, mood disturbance, and body image concerns associated with cancer treatment.

### Gaps in menopause care for cancer survivors

Despite the significant symptom burden associated with cancer treatment-induced menopause, substantial gaps remain in counselling, access to specialist care, and symptom management among cancer survivors. Many women report receiving limited information beyond cancer risk reduction, with issues such as sexual health, cardiovascular risk, and body image often under-discussed. Consequently, survivors enter menopause with insufficient knowledge or support, amplifying distress and impairing quality of life. This is reflected in patient-reported survivorship data, in which menopause symptoms and associated health changes were reported to significantly impact quality of life in 86%, whilst 95% reported not receiving adequate support from their healthcare team during or after cancer treatment (Talaulikar *et al.* 2025).

Access to specialised menopause care is limited, with care commonly fragmented across specialities, primary care, and self-management (Peate *et al.* 2021). Effective therapies are often underutilised due to lack of resources, clinician uncertainty, concerns regarding oncological safety, and inconsistent guidance (Yongue *et al.* 2023). In the VIVROVAIRE study of ovarian cancer survivors, only 5 of 59 women (8.5%) received HRT despite 76% having no clear contraindication (Gernier *et al.* 2021). Additionally, patient-reported barriers, such as concerns about treatment efficacy, treatment cost, stigma, and inadequate clinician knowledge about treatment options (Hirschberg *et al.* 2025), further restrict timely intervention.

A key structural contributor to these gaps is the limited recognition of cancer treatment-induced menopause as a distinct clinical entity within existing healthcare frameworks. Menopause following cancer treatment is often subsumed within general menopause guidance, without adequately addressing the specific risks and survivorship needs of this population. This lack of tailored guidance has implications for service provision and care pathways. However, it is also important to recognise that whilst some patients require specialist multidisciplinary input, a substantial proportion can be effectively supported within current services, provided that clinicians are equipped with appropriate knowledge and resources.

## Management principles

Management of menopause following cancer treatment requires an individualised, risk-stratified approach that balances oncological safety with quality-of-life priorities and prevention of future morbidity. As symptoms are driven by both menopause and oncological processes, HRT alone often does not adequately address the full spectrum of symptom burden, and a multimodal management approach is required. Figure 1 provides a practical framework to guide clinical decision-making, integrating key considerations including oncological risk, symptom burden, and long-term health outcomes.

**Figure 1:**
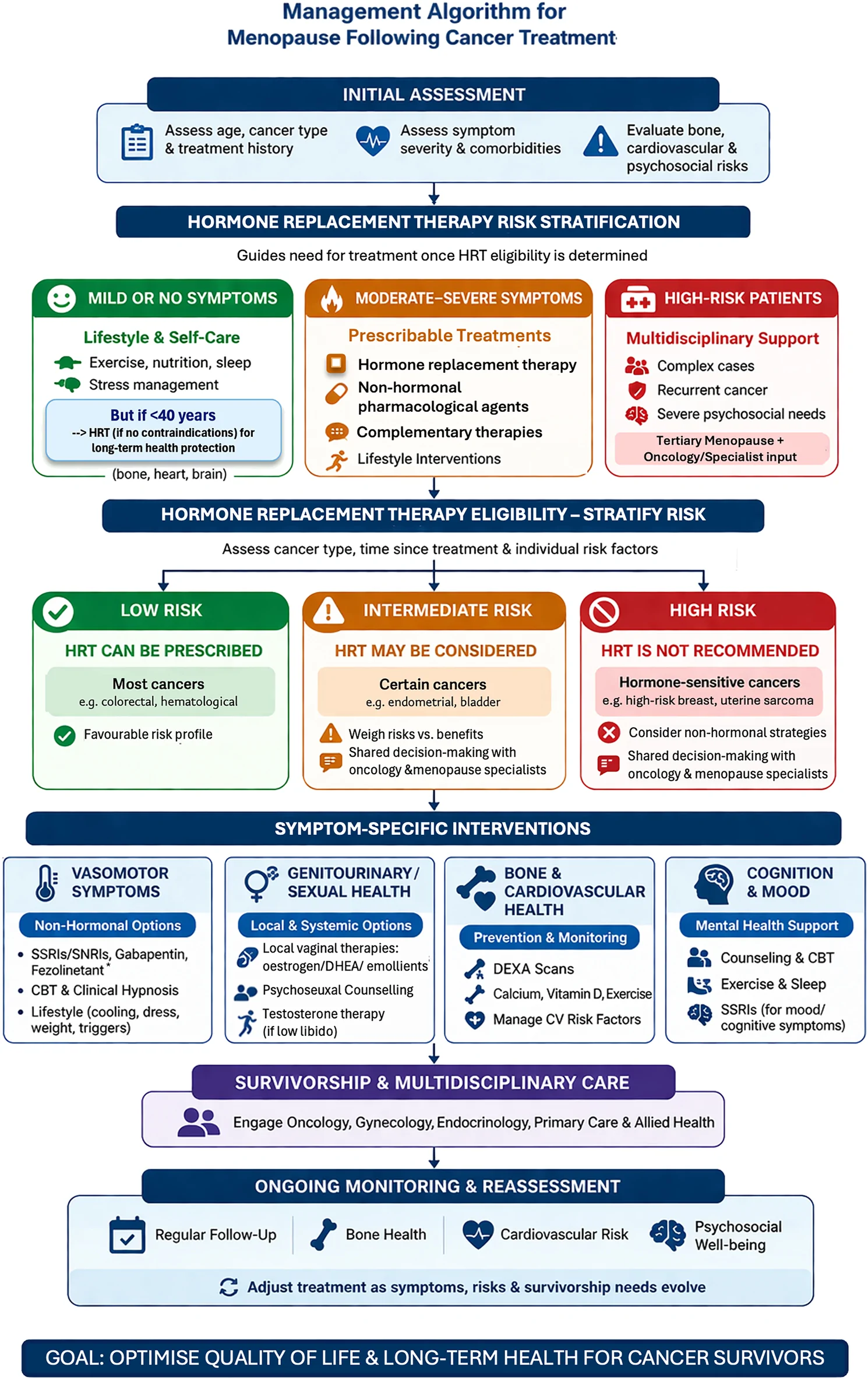
Algorithm outlining a stepwise, individualised approach to the management of menopause following cancer treatment-induced menopause. *Neurokinin-3 receptor antagonists are not currently approved by NICE for use in breast cancer populations, with guidance pending results from ongoing trials (including the HIGHLIGHT study). However, fezolinetant is now available on NHS prescription for non-oncological populations. SSRIs, selective serotonin reuptake inhibitors; SNRIs, serotonin–norepinephrine reuptake inhibitors; CBT, cognitive behavioural therapy; DEXA, dual-energy X-ray absorptiometry; CV, cardiovascular.

### Hormone replacement therapy recommendations

HRT remains the most effective treatment for menopausal symptoms and for the prevention of long-term sequelae of oestrogen deficiency in those with premature/early menopause. However, its use in cancer survivors requires careful consideration according to recurrence risk, tumour biology (see Fig. 2), and individual patient factors and preferences.

In women at low oncological risk, HRT can be prescribed and should be actively considered, particularly in those with POI. In this group, treatment is indicated not only to improve symptoms but also to mitigate the risks of long-term morbidity. HRT is recommended up until the average age of natural menopause (51 years) at which point its risks and benefits should be re-evaluated.

In women at intermediate risk, HRT may be considered following an individualised risk–benefit assessment. Non-hormonal treatments should be explored in the first instance; however, if they are not effective or there is patient preference, then HRT can be considered.

In women at high risk, systemic HRT is generally not recommended. In these patients, management should prioritise non-hormonal strategies and targeted local therapies where appropriate. For some women, an individualised multidisciplinary approach to HRT may be required if symptoms are severe and non-hormonal options are not acceptable or effective.

### Prescribing HRT

When prescribing HRT for women with cancer treatment-induced menopause, it is important to recognise that this population often carries a higher baseline risk profile. Oral oestradiol is an effective option and may be used in appropriately selected women. However, transdermal oestrogen is recommended in those receiving active cancer treatment or with comorbidities, such as an increased risk of venous thromboembolism (Vinogradova *et al.* 2019). Additionally, oral oestradiol should be used with caution in women at risk of drug interactions, such as those receiving systemic anticancer therapies, or in those with gastrointestinal malabsorption (e.g. radiation enteritis), as absorption may be unreliable.

Women with an intact uterus, including those who have undergone pelvic radiotherapy, should be prescribed an adequate dose of progesterone/progestogen for endometrial protection. This should also be considered in hysterectomised women with a history of endometrial cancer, or endometriosis where there is a theoretical risk of stimulating residual disease. The choice of progestogen may also be clinically relevant. Limited evidence suggests that micronized progesterone and dydrogesterone may have a more favourable breast safety profile in low-risk women (Fournier *et al.* 2007). Therefore, they may also be preferred in those with a history or increased risk of breast cancer, including BRCA mutation carriers. Off-label use of vaginal progesterone should be avoided in women who have undergone vaginal radiotherapy due to the risk of poor absorption.

## Special considerations in hormone replacement therapy

### Tibolone

Tibolone is a selective tissue regulator with oestrogenic, progestogenic, and androgenic activity and has demonstrated efficacy comparable to conventional HRT. Due to its weak oestrogenic activity, it is less effective at managing severe symptoms in POI. However, it does effectively improve BMD and reduce fracture risk (HR: 0.73; 95% CI: 0.59–0.92) (Barrionuevo *et al.* 2019). It may also offer a lower venous thromboembolism risk (RR: 0.92; 95% CI: 0.77–1.10 relative to non-use) compared with oral oestradiol (RR: 1.54; 95% CI: 1.44–1.65) (Renoux *et al.* 2010) and therefore provide an alternative option for poor transdermal absorbers. Although tibolone appears to have a less stimulatory effect on breast tissue and does not increase mammographic density, its oncological safety continues to be uncertain. The LIBERATE trial was terminated early due to increased risk of breast cancer recurrence in those with oestrogen receptor-positive tumours (HR: 1.56, 95% CI: 1.22–2.01, *P* = 0.0005) (Kenemans *et al.* 2009). Consequently, tibolone cannot be assumed to be a safer alternative and should not be prescribed where standard HRT is contraindicated.

### Androgen therapy

Transdermal testosterone therapy at physiological doses for premenopausal women improves sexual function in hypoactive sexual desire disorder (HSDD) when other causes have been excluded and baseline serum levels are low. Current evidence does not support its use for cognition, mood, or musculoskeletal outcomes (e.g. BMD and muscle strength). However, trials such as the ESTEEM study are anticipated to address some of these evidence gaps (ISRCTN Registry 2025). Epidemiological studies have demonstrated that increased endogenous testosterone may be associated with increased breast cancer risk (HR: 1.18; 95% CI: 1.14–1.23) (Tin Tin *et al.* 2021). In contrast, a randomised, double-blind, placebo-controlled trial involving 250 women, with no history of breast cancer, reported that exogenous testosterone therapy did not significantly increase mammographic breast density, and short-term use was not associated with increased breast cancer incidence (Davis *et al.* 2009). However, the follow-up was only 52 weeks, which limits the ability of this study to establish the long-term breast safety of testosterone. A single-arm small pilot study of 29 breast cancer survivors undergoing ovarian suppression and aromatase inhibitor therapy evaluated a 3-month course of low-dose testosterone and demonstrated maintenance of baseline oestradiol levels alongside significant improvements in sexual function scores (Taranto *et al.* 2024). Long-term safety was not a reported outcome, and larger, longitudinal randomised controlled trials are required to assess this.

As there is limited information on the oncological safety of testosterone, particularly after hormone-positive cancers, such as breast cancer, and concerns over the peripheral conversion of testosterone to oestradiol, testosterone is not recommended.

### Modifying endocrine therapy

Tamoxifen and aromatase inhibitors are associated with menopausal symptoms. Symptoms typically become apparent within 3–6 months of treatment, after which they often stabilise or improve (Marsden 2016). Switching endocrine therapies may help alleviate specific adverse effects (such as arthralgia and hair loss) but should only be considered in consultation with the oncology team.

### Combined use of HRT with selective oestrogen receptor modulators

The use of HRT with selective oestrogen receptor modulators (SERMs) is not routinely recommended. In selected cases if HRT is considered, switching from an aromatase inhibitor to tamoxifen may provide theoretical reassurance, as tamoxifen antagonises oestrogen receptors in breast tissue. Although bazedoxifene combinations show some favourable breast safety in the general population, they remain contraindicated after breast cancer.

### Symptom management using non-hormonal therapies

Non-hormonal therapies play a key role in managing cancer treatment-induced menopause, especially in those for whom HRT is contraindicated or declined. However, their value extends beyond this subgroup of patients. As previously outlined, symptoms in cancer survivors often coalesce, due to the combined effects of endocrine changes, disease factors, and treatment-related toxicities. Therefore, HRT alone may not be sufficient to address the full range of symptoms or to improve quality of life. Also, confidence in counselling women on non-hormonal therapies facilitates patient choice.

Non-hormonal approaches can be used to target individual symptoms. These include pharmacological agents, behavioural and psychological interventions, and lifestyle-based strategies, each with variable efficacy. A tailored approach should prioritise the most burdensome symptoms, whilst accounting for comorbidities, concurrent cancer treatments, and patient preferences.

### Pharmacological

Pharmacological therapies (Table 2) can be less effective than HRT if used individually. Their use for managing menopause is off-label, except for clonidine and neurokinin receptor antagonists. Most prescribable agents target vasomotor symptoms, with individual selective benefits for mood (antidepressants), pain or arthralgia (gabapentinoids), and urinary symptoms (oxybutynin). As such, by providing targeted symptom control, they can achieve improvements in quality of life without complete menopause symptom resolution. However, for many women, the side effect profile of these agents outweighs the benefits of these agents, and longer-term complications, such as sexual dysfunction with the SSRIs and addiction potential (SSRIs/gabapentinoids), should be discussed before initiating treatment. Drug interactions must also be considered, particularly cytochrome P4502D6 inhibition in tamoxifen users.

**Table 2:** Non-hormonal therapies for vasomotor symptoms: efficacy, safety, and considerations in oncology populations.

Class/agent	Typical dose	Efficacy (vs placebo)	Key adverse effects	Tamoxifen interaction/oncology considerations
SSRIs/SNRIs		∼10–25% reduction (up to 40–65% with paroxetine/venlafaxine)	Drowsiness, weight gain, sexual dysfunction, hypertension	Avoid: paroxetine, fluoxetine (strong CYP2D6 inhibitors)
Paroxetine	7.5 mg			
Citalopram	10–30 mg			
Escitalopram	10–20 mg			
Venlafaxine	37.5–150 mg			
Neurokinin receptor antagonists		∼20–25% reduction	Hepatotoxicity (requires LFT monitoring), fatigue, headache	Elinzanetant studied in breast cancer (OASIS-4) and appears safe; fezolinetant not studied in cancer population (off-label use)
Fezolinetant	45 mg			
Elinzanetant	120 mg			
Gabapentinoids		∼10–25% reduction (higher doses approach oestrogen efficacy)	Sedation, dizziness, addiction potential	No CYP450 interaction
Gabapentin	300–900 mg/day			
Pregabalin	75–150 mg BD			
Antimuscarinics		∼30–50% reduction	Dry mouth, constipation, cognitive impairment	No CYP2D6 interaction; caution in older patients due to cognitive effects
Oxybutynin	2.5–5 mg BD			
Antihypertensives		∼10–20% reduction	Hypotension, dry mouth, sedation, rebound hypertension	No CYP interaction
Clonidine	25–75 μg/day			

SSRIs, selective serotonin reuptake inhibitors; SNRIs, serotonin–norepinephrine reuptake inhibitors.

Other pharmacological agents may offer selective symptom benefit. For example, mirtazapine can improve sleep and mood, although its use is limited by side effects, including weight gain, sedation, and cognitive effects. Daridorexant, approved for chronic insomnia where non-pharmacological measures have been insufficient, reduces nocturnal awakenings without significant daytime sedation. It should only be used as a short-term intervention with appropriate monitoring and coordination with primary care.

## Non-pharmacological

### Cognitive behavioural therapy

Cognitive behavioural therapy (CBT) is an evidence-based intervention grounded in a biopsychosocial model of menopause. Randomised controlled trials, including MENOS-2 (Ayers *et al.* 2012), have demonstrated significant and sustained reductions in vasomotor symptoms (50%) and improvements in sleep and quality of life with the use of either group or self-help CBT.

### Lifestyle interventions

Lifestyle interventions provide modest but clinically meaningful benefits in both menopause symptoms and longer-term health. Practical measures, such as smoking cessation, reducing alcohol and caffeine intake, avoiding triggers and cooling strategies, alleviate vasomotor symptoms. Mind–body interventions (e.g. yoga, Pilates, and Tai Chi) may improve musculoskeletal symptoms, emotional well-being, and energy levels. Emerging evidence links diet and microbiome, with reduced intake of saturated fats and ultra-processed foods linked to improvements in cognition and mood (Cuozzo *et al.* 2026). Additionally, optimisation of modifiable risk factors can be beneficial for symptom management. For example, improving metabolic health can support cognitive function, whilst weight loss may improve both arthralgia and vasomotor symptoms (Anekwe *et al.* 2025).

### Over-the-counter supplements

A wide range of over-the-counter supplements (Table 3) are commonly used by women to manage menopausal symptoms despite variable and often limited evidence of activity. Some may have oestrogenic effects or interact with cancer therapies and therefore careful counselling regarding safety and efficacy is required.

**Table 3:** Over-the-counter supplements for menopausal symptoms: efficacy and safety considerations (Mullin & Manley 2025).

Supplement	Indications/effects	Evidence summary	Key safety considerations
Ashwagandha	QoL, cognition	Improves QoL; may increase oestradiol	Uncertain safety in hormone-sensitive cancers
Black cohosh	VMS, mood, sleep	Comparable to low-dose oestradiol (short term)	Limited long-term safety; caution in hormone-sensitive cancers
Evening primrose oil	Psychological symptoms	Limited RCT evidence for mood improvement	Interacts with anticoagulants
Ginkgo biloba	Cognition	Minimal benefit in RCTs	Avoid with anticoagulants
Ginseng (Panax)	VMS, QoL	May improve symptoms; heterogeneous evidence	Oestrogenic; avoid in hormone-sensitive cancers
Glucosamine	Joint pain	Effective for osteoarthritis	No menopause-specific evidence
Lion’s mane	Cognition, mood	Limited but promising evidence	Insufficient menopause-specific data
Maca	Energy, cognition	Insufficient evidence	Limited safety data
Magnesium	Sleep, anxiety	Low-quality evidence for improved sleep and anxiety	Limited high-quality data
Melatonin	Sleep, mood	Improves sleep onset; possible mood benefit	Caution in autoimmune conditions
Milk thistle	VMS	Reduces flush frequency/severity	Possible oestrogenic activity; drug interactions; limited safety in cancer
Omega-3 fatty acids	VMS	Reduce VMS (when taken with vitamin E)	High doses may reduce tamoxifen efficacy
Phyto-oestrogens (soy, red clover)	VMS, possible cancer modulation	No consistent VMS benefit; dietary intake may reduce recurrence	High-dose supplements not recommended in hormone-sensitive cancers
Pollen extract	VMS, mood, sleep	Reduces flushes (∼27%); improves sleep, mood, fatigue	Non-oestrogenic; well tolerated
Sage	VMS	Reduces flush severity (50–64%)	Potential oestrogenic effects; avoid in epilepsy
St John’s wort	Mood	Effective vs placebo; similar to antidepressants	Significant drug interactions (tamoxifen, HRT, PARP inhibitors)
Turmeric (curcumin)	VMS, joint pain	Reduces flushes (by up to 73%); anti-inflammatory	May reduce tamoxifen efficacy; liver toxicity (rare); caution with anticoagulants
Vitamin D	Bone health, inflammation	Supports bone strength; may reduce inflammation	Generally safe
Vitamin E	VMS	Moderate reduction in VMS (when taken with omega-3)	High doses may reduce tamoxifen efficacy

QOL, quality of life; VMS, vasomotor symptoms; RCT, randomised controlled trial.

### Management of genitourinary syndrome of menopause

Low-dose topical vaginal oestrogen is the most effective therapy for GSM with minimal systemic absorption. As such, it is recommended by the National Institute for Health and Care Excellence as the first-line management of GSM (National Institute for Health & Care Excellence 2025). The main exception to this guidance is for those with breast cancer using an aromatase inhibitor (see also Fig. 2). For this subgroup, non-hormonal moisturisers and lubricants should be offered as a first-line treatment with escalation to vaginal oestrogen on an individualised basis. Furthermore, switching to tamoxifen may permit safer use but must be balanced against oncological efficacy (Baumgart *et al.* 2013). Where vaginal oestrogen is considered, the preparation choice may be relevant. Oestriol is a weaker oestrogen with a preferential affinity for urogenital rather than breast oestrogen receptors (Katzenellenbogen 1984) and does not convert to the more potent oestradiol *in vivo*. In women with persistent symptoms, consider irritant reactions or alternative diagnoses, such as vulval dermatoses, radiotherapy changes, or graft-versus-host disease, after allogenic stem cell transplants.

**Figure 2:**
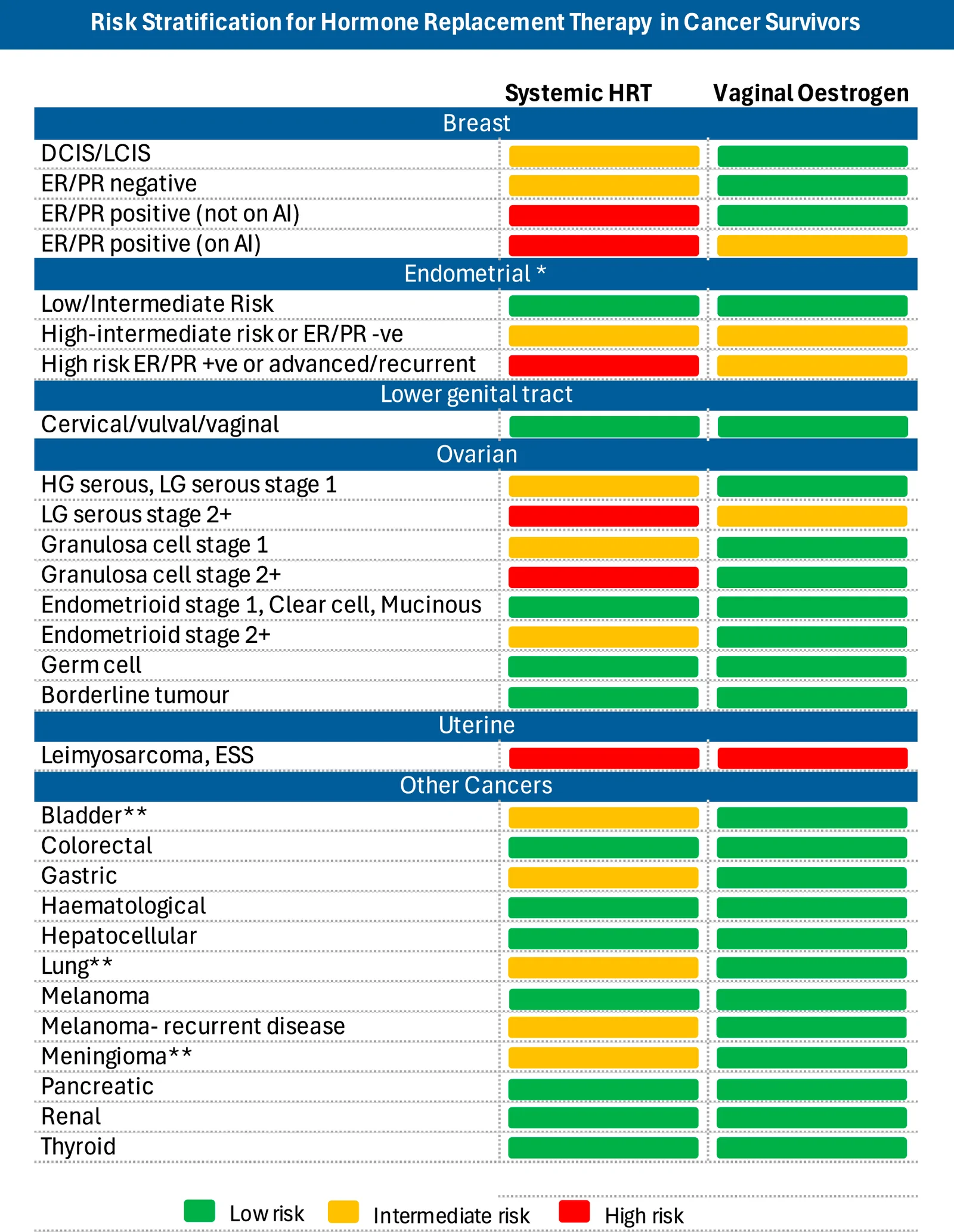
Risk stratification for hormone replacement therapy in cancer survivors. Risk categories are derived from available evidence, including British Gynaecological Cancer Society recommendations for gynaecological cancers (Taylor *et al.* 2024) and expert guidance (Hickey *et al.* 2024, Yongue *et al.* 2026). *Endometrial cancers are stratified using ESGO prognostic risk groups. **Inconsistent results in studies, some showing increased risk whilst others do not. DCIS, ductal carcinoma *in situ*; LCIS, lobular carcinoma *in situ*; AI, aromatase inhibitor; HG, high grade; LG, low grade; ESS, endometrial stromal sarcoma.

Alternative treatments for GSM include ospemifene and vaginal DHEA. Ospemifene is an oral SERM that improves vaginal dryness and dyspareunia by up to 80% at 12 months (Di Donato *et al.* 2019). It can be useful when vaginal treatment is impractical. However, there are no safety data for its use in hormone-sensitive cancers. Similarly, prasterone, a vaginal dehydroepiandrosterone (DHEA) insert, is approved for moderate-to-severe dyspareunia in menopause. In a randomised controlled trial of 464 women with prior breast or gynaecological cancer, it improved symptoms and sexual function without increasing serum oestradiol levels (Barton *et al.* 2018).

### Models of care

The management of menopause after cancer requires a multidisciplinary approach due to the complex and evolving needs of cancer survivors, which are shaped by disease characteristics, treatment exposures, comorbidities, and individual preferences. Despite this, access to specialist menopause services remains limited, and many clinicians report uncertainty on how to apply existing evidence to clinical practice (Yongue *et al.* 2023).

Multidisciplinary menopause and survivorship services offer a strategy to address these challenges, by offering expertise from oncology, gynaecology, endocrinology, primary care, and allied health. An example of this type of approach is the Menopausal Service after Cancer (MSAC) in Australia. This service is reported to have resulted in significant improvements in patient-reported outcomes, including vasomotor symptoms, fatigue, sleep disturbance, and sexual well-being (Hollingworth *et al.* 2022). However, such resource-intensive models may not be feasible in all healthcare settings.

A more practical approach is through the integration of menopause care within routine oncology pathways. Given the frequency of oncology follow-up, embedding menopause assessment and management into cancer care, supported by multidisciplinary discussions, supports the provision of individualised, evidence-based care whilst continuing access to specialist input (Brierley *et al.* 2026). Mind–body interventions, lifestyle modification, and non-pharmacological therapies can help improve overall well-being whilst complementing conventional treatments. Digital health initiatives have also been shown to improve symptoms, increase education, and empower patients with early menopause (Yeganeh *et al.* 2022).

A priority for future service development is the establishment of consensus across societies that recognise cancer treatment-induced menopause as a distinct clinical entity. There also needs to be clear guidance that delineates suitable care pathways. Existing models currently do not distinguish between patients who can be managed effectively in primary care and those requiring specialist or tertiary input. A stratified approach may help guide referral pathways and optimise resource utilisation to ensure that specialist services are targeted to those with the greatest need.

### Future research agenda

Despite increasing recognition of cancer treatment-induced menopause as a significant survivorship issue, there are significant evidence gaps. The international Menopause Priority Setting Partnership (MAPS) (Nash *et al.* 2024) and the National Institute for Health and Care Excellence (National Institute for Health & Care Excellence 2025) have both identified key areas requiring further investigation. These include the identification of safe and effective non-hormonal therapies, particularly for women in whom HRT is contraindicated; optimisation of management strategies in breast cancer populations, where uncertainty regarding oncological safety persists; and clarification of the long-term safety, duration, and optimal regimens of hormone replacement therapy in higher-risk groups. In addition, high-quality longitudinal studies evaluating the safety of low-dose vaginal oestrogen in women using aromatase inhibitors and testosterone after breast cancer would also be of benefit.

## Conclusion

Menopause induced by cancer treatment presents a unique and complex clinical challenge that extends beyond the natural physiological process of menopause. Its abrupt onset, combined with the effects of oncological therapies, results in a multidimensional burden encompassing physical, psychological, and social domains.

Although there is a growing awareness of cancer treatment-induced menopause as a significant survivorship issue, gaps remain in access to care, clinician education, and integration of menopause management within care pathways. Therefore, multidisciplinary and scalable models of care are required to address this unmet need. Such models would utilise specialist menopause services, integrate oncology pathways, and employ digital health interventions. Through a coordinated, patient-centred approach, survivorship and menopause care would balance both oncological safety and quality-of-life priorities.

Future research should focus on the safety and efficacy of therapeutic interventions in cancer populations, including hormone replacement therapy, novel non-hormonal agents, and integrative approaches. Additionally, greater emphasis is required on translating existing evidence into routine clinical practice.

Ultimately, improving outcomes for women with menopause induced by cancer treatment requires a shift away from the reactive management of symptoms and towards a more proactive integrative model that recognises menopause as an important component of cancer survivorship rather than a secondary consequence of treatment.

## Declaration of interest

SN declares receiving consulting fees from GSK, AbbVie, Bayer, and Pharma&; payment for lectures/presentations from GKS, AbbVie, and Pharma&; and travel expenses for attending meetings from MSD, GSK, and AbbVie; is on advisory boards at GSK, Biotech, Tubulis, Regeneron, Immunocore, AbbVie, Bayer, Pharma&; and has stock ownership in GSK, AstraZeneca, and Roche Family.

## Funding

This research did not receive any specific grant from any funding agency in the public, commercial, or not-for-profit sector.

## Author contribution statement

GY drafted the manuscript. ZN, KM, MD, and SN contributed with significant edits to the final version.
